# Impaired Osteogenesis of Disease-Specific Induced Pluripotent Stem Cells Derived from a CFC Syndrome Patient

**DOI:** 10.3390/ijms18122591

**Published:** 2017-12-01

**Authors:** Jung-Yun Choi, Kyu-Min Han, Dongkyu Kim, Beom-Hee Lee, Han-Wook Yoo, Jin-Ho Choi, Yong-Mahn Han

**Affiliations:** 1 Graduate School of Medical Science and Engineering, Korea Advanced Institute of Science and Technology (KAIST), Daejeon 34141, Korea; alice_choi@kaist.ac.kr; 2 Department of Biological Sciences, KAIST, Daejeon 34141, Korea; hankm88@kaist.ac.kr (K.-M.H.); dkkim86@gmail.com (D.K.); 3 Department of Pediatrics, Asan Medical Center Children’s Hospital, University of Ulsan College of Medicine, Seoul 05505, Korea; bhlee@amc.seoul.kr (B.-H.L.); hwyoo@amc.seoul.kr (H.-W.Y.); jhc@amc.seoul.kr (J.-H.C.)

**Keywords:** cardiofaciocutaneous syndrome, induced pluripotent stem cells, bone development, mesenchymal stem cells, Osteoblasts, ERK, bone morphogenetic protein (BMP), transforming growth factor-β (TGF-β), SMAD1, SMAD2

## Abstract

Cardiofaciocutaneous (CFC) syndrome is a rare genetic disorder caused by mutations in the extracellular signal-regulated kinase (ERK) signaling. However, little is known about how aberrant ERK signaling is associated with the defective bone development manifested in most CFC syndrome patients. In this study, induced pluripotent stem cells (iPSCs) were generated from dermal fibroblasts of a CFC syndrome patient having rapidly accelerated fibrosarcoma kinase B (BRAF) gain-of-function mutation. CFC-iPSCs were differentiated into mesenchymal stem cells (CFC-MSCs) and further induced to osteoblasts in vitro. The osteogenic defects of CFC-MSCs were revealed by alkaline phosphatase activity assay, mineralization assay, quantitative real-time polymerase chain reaction (qRT-PCR), and western blotting. Osteogenesis of CFC-MSCs was attenuated compared to wild-type (WT)-MSCs. In addition to activated ERK signaling, increased p-SMAD2 and decreased p-SMAD1 were observed in CFC-MSCs during osteogenesis. The defective osteogenesis of CFC-MSCs was rescued by inhibition of ERK signaling and SMAD2 signaling or activation of SMAD1 signaling. Importantly, activation of ERK signaling and SMAD2 signaling or inhibition of SMAD1 signaling recapitulated the impaired osteogenesis in WT-MSCs. Our findings indicate that SMAD2 signaling and SMAD1 signaling as well as ERK signaling are responsible for defective early bone development in CFC syndrome, providing a novel insight on the pathological mechanism and therapeutic targets.

## 1. Introduction

Extracellular signal-regulated kinase (ERK) signaling plays an important role in mammalian embryonic development by regulating differentiation, proliferation, and cell cycle [1,2]. Germ-line mutations in ERK signaling are related to a variety of disorders called RASopathies, including neurofibromatosis type-1, LEOPARD syndrome (an acronym formed from its main features; that is, lentigines, electrocardiographic abnormalities, ocular hypertelorism, pulmonary valve stenosis, abnormal genitalia, retardation of growth and deafness), Noonan syndrome, Costello syndrome, and cardiofaciocutaneous (CFC) syndrome [3,4]. CFC syndrome, an autosomal dominant disorder, is mostly caused by gain-of-function mutations in the *BRAF* gene [5,6]. Gain-of-function of BRAF, a serine/threonine-specific protein kinase, results in downstream ERK activation in the ERK signaling pathway. The representative symptoms of CFC syndrome include cardiac disorder, facial malformation, cutaneous anomaly, short stature, and mental retardation [7]. Among them, no specific treatment has been developed for skeletal defects, including short stature, bone growth delay, low bone mineral density, short neck, craniofacial malformation, kyphosis, scoliosis, and funnel chest, though the majority of CFC syndrome patients suffer from diverse skeletal defects [8]. Although *BRAF*-mutant mice show several skeletal defects [9], they do not completely recapitulate the phenotypes of CFC syndrome patients because of interspecies differences.

Mammalian skeletal development begins with the condensation of mesenchymal stem cells (MSCs) [10]. During skeletal development, osteogenic differentiation entails extracellular matrix mineralization, which is a complicated process to form hydroxyapatite (HA) [11]. HA is mainly composed of calcium and phosphate, and the formation of HA is tightly regulated by various factors. For HA synthesis, alkaline phosphatase (ALP) hydrolyses pyrophosphate (PPi) to inorganic phosphate, ectonucleotide pyrophosphatase/phosphodiesterase 1 (ENPP1) synthesizes PPi, and ankylosis human (ANKH) transports PPi to the extracellular matrix where HA is formed [12]. In addition, osteopontin (OPN) inhibits HA crystal formation, whereas bone sialoprotein (BSP) promotes initiation of HA crystal formation [13,14]. Although the roles of these factors are in part understood in bone matrix mineralization, little is known about how these factors influence the bone mineralization process in CFC syndrome.

In addition, various growth factors and cytokines, such as bone morphogenetic protein (BMP), transforming growth factor-β (TGF-β), fibroblast growth factor, Notch, Wingless-related integration site, Sonic hedgehog, and parathyroid hormone-related peptide are associated with skeletal development [15]. In particular, TGF-β signaling and BMP signaling are known to play important roles in bone formation during mammalian development [16]. Disruption of Tgfbr2 leads to defective long bone and joint development in mice [17], and downregulation of Bmp signaling results in abnormal endochondral ossification and osteoporosis in mice [18,19]. However, it is not known whether TGF-β and BMP signaling are associated with aberrant bone development in CFC syndrome patients.

In this study, it is suggested that an increased SMAD2 pathway which responds to TGF-β receptors may play an important role in defective bone development in CFC patients during osteogenesis. CFC syndrome-specific induced pluripotent stem cell (iPSC)-derived MSCs exhibited low ALP activity, insufficient mineral deposition, and abnormal expression of osteogenic genes during osteogenic differentiation in vitro. Intriguingly, an enhanced SMAD2 pathway caused by activated ERK signaling was observed in CFC-MSCs. Moreover, we found that the activated SMAD2 pathway decreased the SMAD1 pathway in CFC-MSCs during osteogenesis. The SMAD1 pathway responds to BMP receptors. Notably, treatment with ERK signaling inhibitor, a SMAD2 pathway inhibitor, and SMAD1 pathway agonist recovered abnormal phenotypes in CFC-MSCs during osteogenic differentiation. These results indicate that a downregulated SMAD1 pathway caused by an enhanced SMAD2 pathway may account for impaired osteogenesis in CFC syndrome during early development.

## 2. Results

### 2.1. Generation and Characterization of CFC-iPSCs

The patient from which fibroblasts were harvested showed typical symptoms of CFC syndrome, including cardiac disorder, facial malformation, cutaneous anomaly, short stature, and mental retardation (Table S1). Even though the patient has been treated with human growth hormone, defects of his skeletal developmental marks such as height and bone mineral density have not been alleviated (Table S1). To study the pathological mechanisms, CFC-iPSCs were generated from dermal fibroblasts of the CFC syndrome patient with *BRAF* Q257R (c. 770A > G), which is the most frequent mutation found in CFC syndrome [20]. The two lines of CFC-iPSCs (CFC2 and CFC7) employed in this study were characterized. In brief, pluripotency of iPSCs was confirmed by immunostaining of pluripotency marker proteins, formation of EBs with three germ layer cells, and in vivo teratoma formation. CFC-iPSCs exhibited no expression of transgenes and normal karyotypes. *BRAF* mutation was identified in CFC-fibroblasts and iPSCs (Figure S1). Thus, CFC-iPSCs were successfully generated.

### 2.2. Impaired Osteogenesis of MSCs Derived from CFC-iPSCs In Vitro

**Figure 1:**
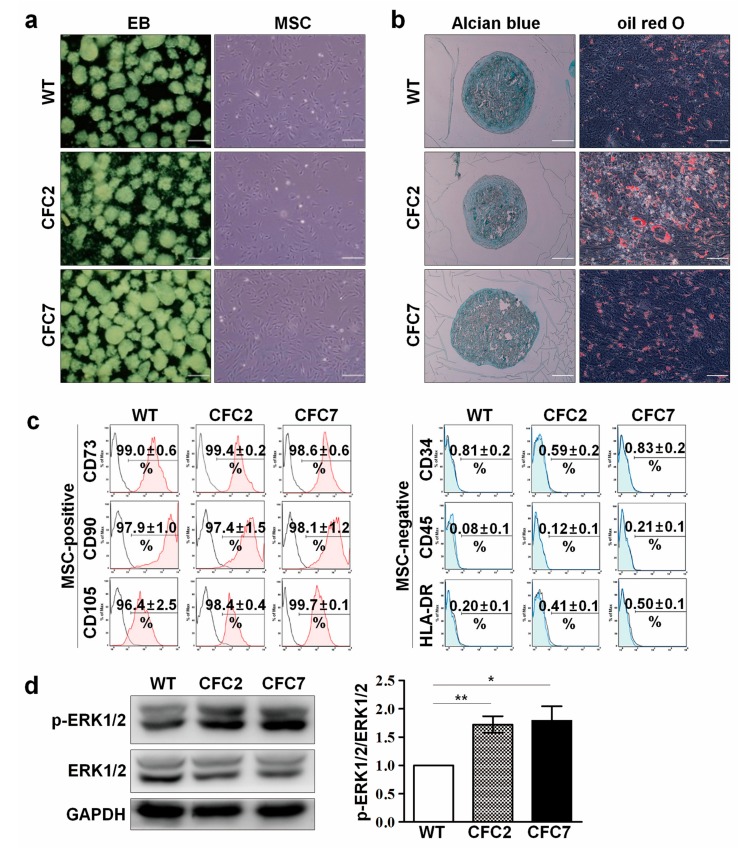
Induction of iPSCs into MSCs. (**a**) Normal differentiation of EBs and MSCs developed from CFC-iPSCs. WT- and CFC-derived EBs (left panel) and MSCs (right panel) had a normal morphology. Scale bars, 200 μm; (**b**) Differentiation of CFC-MSCs into chondroblasts (alcian blue staining, left panel) and adipocytes (oil red O staining, right panel). Scale bars, 500 μm (left panel) and 50 μm (right panel); (**c**) Expression profiles of MSC-positive and -negative surface markers; (**d**) Increased p-ERK1/2 level in CFC-MSCs compared to WT-MSCs. The band intensity of CFC-MSCs was measured using Image J, normalized to that of GAPDH, and expressed as fold changes relative to that of WT-MSCs. Data were described as the mean ± SEM (n = 3, biological replicate). * *p* < 0.05, ** *p* < 0.01.

Overall protocols for differentiation of human iPSCs into osteoblasts (Obs) are described in Figure S2. The attached CFC-EBs developed normally into MSCs that exhibited a morphology similar to that of WT-EBs (Figure 1a). Recommended criteria of in vitro-derived MSCs generally encompass plastic adherence, typical expression pattern of surface markers, and differentiation potential into three mesenchymal cell types (Obs, chondroblasts, and adipocytes) in vitro [21]. CFC-MSCs easily adhered to plastic culture dishes and exhibited high expression (more than 95%) of positive surface markers (CD73, CD90, and CD105) and no expression of negative surface markers (CD34, CD45, and HLA-DR) (Figure 1c), indicating normal induction of CFC-iPSCs to MSCs. Like WT-MSCs, CFC-MSCs differentiated normally into chondroblasts and adipocytes (Figure 1b). To determine whether the iPSC-derived MSCs retained activated ERK signaling due to *BRAF* mutation, the protein level of p-ERK was examined. As expected, the relative intensity of p-ERK was significantly elevated in CFC-MSCs compared to WT-MSCs (Figure 1d).

**Figure 2:**
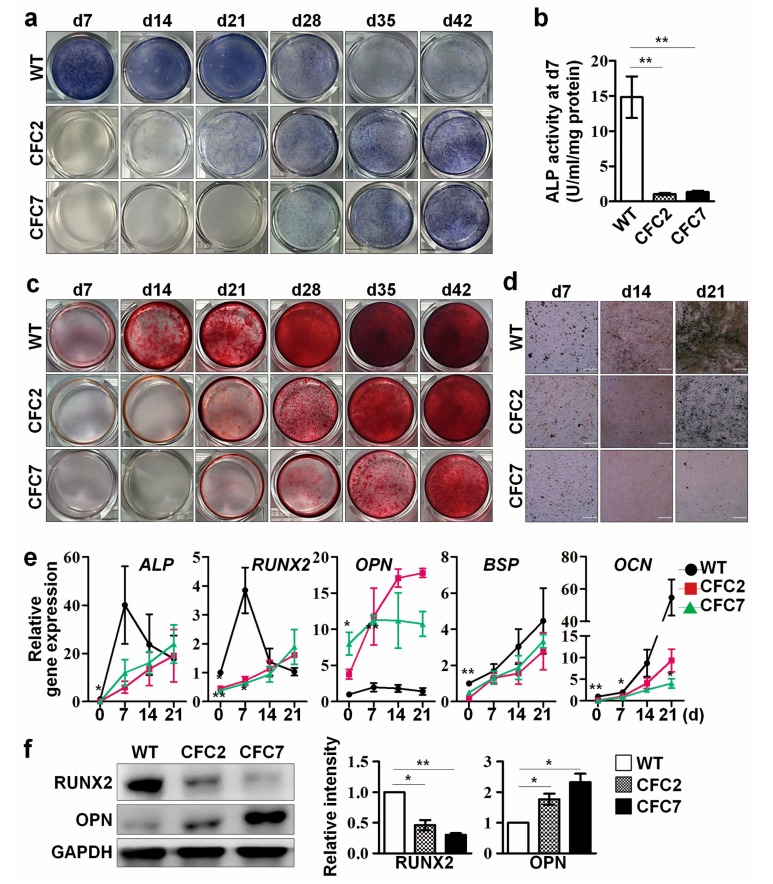
Defects in osteogenic differentiation of CFC-MSCs. (**a**) Representative images of ALP activity in WT- and CFC-MSCs during osteogenic differentiation. ALP activity is shown in blue. Delayed ALP activity was observed in CFC-MSCs during osteogenesis; (**b**) Low ALP activity in CFC-MSCs during osteogenic differentiation compared to WT-MSCs; (**c**) Representative images of alizarin red S staining in WT- and CFC-MSCs during osteogenic differentiation. Alizarin red S staining is shown in red. Reduced calcium deposition was observed in CFC-MSCs during osteogenesis. CFC-MSCs represented delayed calcium deposition during osteogenesis compared to WT-MSCs; (**d**) Representative images of von Kossa staining in WT- and CFC-MSCs during osteogenic differentiation. Von Kossa staining is shown in black. Reduced calcium deposition was observed in CFC-MSCs during osteogenesis. Scale bars, 200 μm; (**e**) Abnormal transcriptional expression of *ALP*, *RUNX2*, *OPN*, *BSP*, and *OCN* in CFC-MSCs during osteogenic differentiation. The transcription level of each gene was normalized to that of WT-MSCs at d0; (**f**) Downregulation of RUNX2 and upregulation of OPN in CFC-Obs at d7 of osteogenic induction. The band intensity of CFC-MSCs was measured using Image J, normalized to that of GAPDH, and expressed as fold changes relative to that of WT-MSCs. All data were expressed as the mean ± SEM (n = 3, biological replicate). *p < 0.05, ** *p* < 0.01. d, days after osteogenic induction.

However, CFC-MSCs failed to develop to normal Obs. CFC-MSCs showed lower ALP activity than WT-MSCs at early time points (d7, d14, d21 and d28) of osteogenic differentiation (Figure 2a,b). Interestingly, ALP activity was detected at d35 and d42 after osteogenic induction of CFC-MSCs (Figure 2a). This result indicates the possibility that osteogenic development of CFC-MSCs is delayed in vitro. ALP is known to play an important role in generating HA, a major mineral component of bone matrix, by replacing PPi with inorganic phosphate [22]. Therefore, we supposed that decreased ALP activity might lead to abnormal mineralization in CFC-MSCs during osteogenic differentiation. Mineralization capacity was detected in CFC-MSCs during osteogenic differentiation using alizarin red S and von Kossa staining. As expected, consistent with the low ALP activity, CFC-MSCs had poor competence for mineral deposition in the osteogenic process (Figure 2c,d). The transcriptional level of osteogenic genes including *ALP*, *runt-related transcription factor 2 (RUNX2)*, *BSP*, and *osteocalcin* (*OCN*) was lower in CFC-MSCs than in WT-MSCs (Figure 2e). It is reported that the expression of *ALP* and *RUNX2* peaks at the early stage and then reduces at the late stage during osteogenic development [23,24]. Unlike WT-MSCs, transcripts of *ALP* and *RUNX2* gradually increased in CFC-MSCs during osteogenic differentiation, not showing conventional peak pattern (Figure 2e). In the osteogenic process, *BSP* and *OCN* genes showed a low transcriptional level in CFC-MSCs compared to WT-MSCs (Figure 2e). These results indicate that the low expression of osteogenic genes in CFC-MSCs probably accounts for the reduced ALP activity and the decreased mineralization during osteogenic differentiation in vitro. On the other hand, transcription of *OPN*, a potential mineralization inhibitor, was enormously activated in CFC-Obs (Figure 2e). Protein levels of RUNX2 and OPN were consistent with the transcriptional patterns at d7 of osteogenic differentiation between WT- and CFC-MSCs (Figure 2f). Aberrant expressions of RUNX2 and OPN were also detected in CFC-MSCs (Figure S2). These results demonstrate that abnormal expression of osteogenic genes in CFC-MSCs contributes to poor osteogenesis. Therefore, our findings indicate that CFC-MSCs have poor competence for development into Obs in vitro.

### 2.3. Enhanced ERK Signaling Activates SMAD2 Pathway in CFC-MSCs during Osteogenic Differentiation

**Figure 3:**
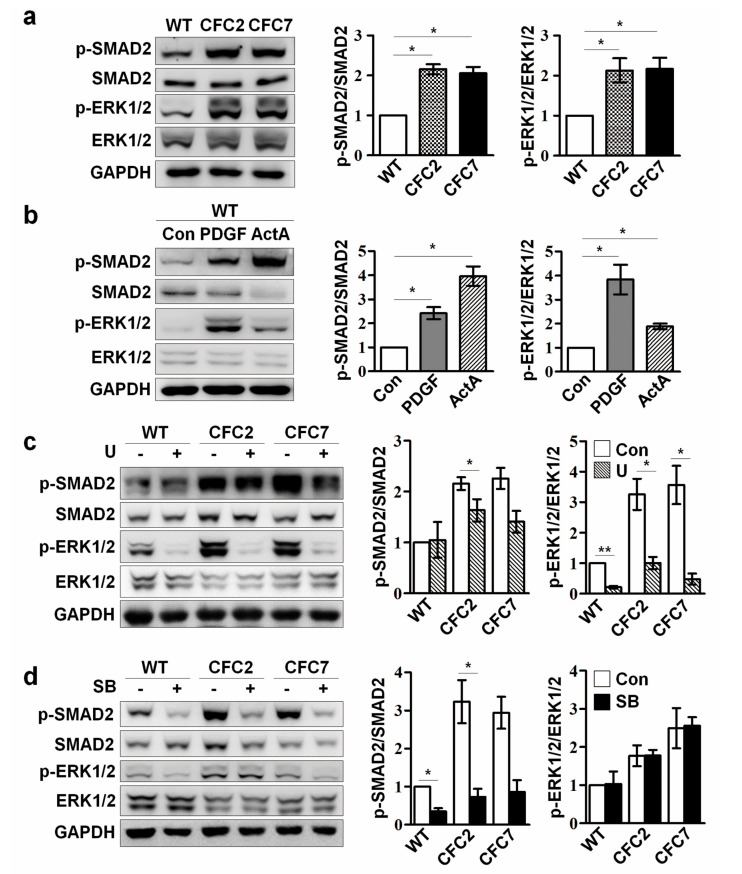
Correlation of ERK signaling and SMAD2 pathway in CFC-MSCs during osteogenic differentiation. (**a**) Activated ERK and SMAD2 pathway in CFC-MSCs during osteogenic differentiation. The levels of p-ERK1/2 and p-SMAD2 were increased in CFC-Obs compared to WT-Obs; (**b**) Regulation of the respective ERK and SMAD2 pathway in the osteogenic process of WT-MSCs. PDGF treatment (10 ng/mL) increased not only p-ERK1/2 but also p-SMAD2, and ActA treatment (50 ng/mL) enhanced not only p-SMAD2 but also p-ERK1/2 in WT-MSCs during osteogenesis; (**c**) Downregulation of both ERK and the SMAD2 pathway in CFC-MSCs by inhibition of ERK signaling. Treatment with U (5 μM) decreased the levels of p-ERK1/2 and p-SMAD2 in CFC-MSCs during osteogenesis; (**d**) Effects of SMAD2 pathway inhibition in CFC-MSCs during osteogenic differentiation. Treatment with a SMAD2 pathway inhibitor (SB-431542; SB; 5 μM) decreased p-SMAD2 level, but did not change p-ERK1/2 level in CFC-MSCs during osteogenesis. Proteins were extracted from respective samples at d7 of osteogenic induction and then subjected to western blot. The band intensity of respective proteins was measured using Image J, normalized to that of GAPDH, and expressed as fold changes. Data were expressed as the mean ± SEM (n = 3, biological replicate). * *p* < 0.05, ** *p* < 0.01.

TGF-β signaling is an important regulator of bone development by which Ob maturation is suppressed [25,26]. Next, experiments were performed to examine whether the osteogenic defects of CFC-MSCs are associated with TGF-β signaling. Enhanced p-SMAD2 as well as p-ERK was observed in CFC-MSCs at d7 of osteogenic induction (Figure 3a). SMAD2 is phosphorylated to form p-SMAD2 by TGF-β receptors. To elucidate whether an enhanced SMAD2 pathway is associated with activated ERK signaling in CFC syndrome, ERK signaling and the SMAD2 pathway was modulated in WT-MSCs during osteogenic differentiation. Activation of ERK signaling by treatment with platelet-derived growth factor-BB (PDGF) led to increments in both p-ERK and p-SMAD2 level, and activation of the SMAD2 pathway by treatment with Activin A (ActA) enhanced both p-ERK and p-SMAD2 level in WT-MSCs during osteogenic differentiation (Figure 3b). Thus, it is conceivable that ERK signaling interacts with the SMAD2 pathway during osteogenesis of MSCs. Next, correlation between ERK signaling or the SMAD2 pathway was examined in CFC-MSCs during osteogenic differentiation. Inhibition of ERK signaling by treatment with U0126 (U) reduced both p-ERK and p-SMAD2 levels in CFC-MSCs during osteogenic differentiation (Figure 3c). In contrast to ERK signaling inhibition, intriguingly, suppression of the SMAD2 pathway did not affect ERK signaling in the osteogenic process of CFC-MSCs (Figure 3d). Collectively, it is suggested that an elevated SMAD2 pathway arising from activated ERK signaling might be a critical regulator of defective osteogenesis in CFC-MSCs.

### 2.4. Enhanced SMAD2 Pathway Downregulates SMAD1 Pathway in CFC-MSCs during Osteogenic Differentiation

**Figure 4:**
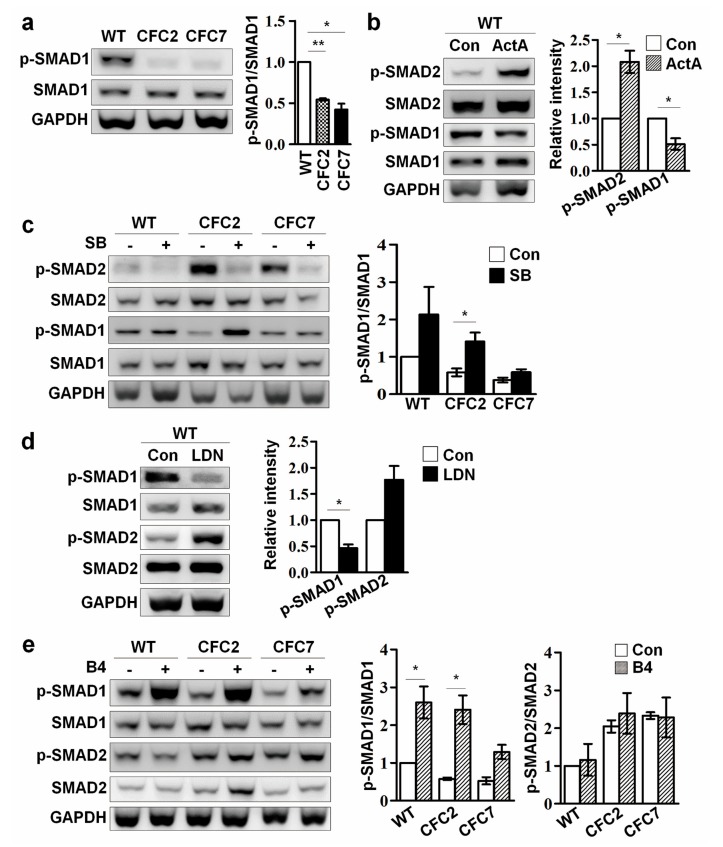
Correlation of SMAD2 and SMAD1 pathways in CFC-MSCs during osteogenic differentiation. (**a**) Reduced level of p-SMAD1 in CFC-Obs compared to WT-Obs; (**b**) Downregulation of the SMAD1 pathway in WT-MSCs during osteogenic differentiation by activation of the SMAD2 pathway. Treatment with ActA (50 ng/mL) significantly reduced the p-SMAD1 level in WT-MSCs during osteogenesis; (**c**) Upregulation of the SMAD1 pathway by SMAD2 pathway inhibition in CFC-MSCs during osteogenic differentiation. Treatment with a SMAD2 pathway inhibitor (SB; 5 μM) increased p-SMAD1 level in CFC-MSCs during osteogenesis; (**d**) Effects of SMAD1 pathway inhibition in CFC-MSCs during osteogenesis. Treatment with LDN (a SMAD1 pathway receptor inhibitor, 100 nM) enhanced the p-SMAD2 level in WT-MSCs during osteogenic differentiation; (**e**) Change in the SMAD2 pathway by a SMAD1 pathway agonist (B4). Treatment with B4 (50 ng/mL) elevated the p-SMAD1 level but did not change the p-SMAD2 level in WT- and CFC-MSCs during osteogenic differentiation. Data were expressed as the mean ± SEM (n = 3, biological replicate). * *p* < 0.05, ** *p* < 0.01.

Along with TGF-β signaling, BMP signaling is also closely involved in bone formation during mammalian development. BMP signaling is known to promote Ob commitment and maturation through activation of downstream factors, including RUNX2, a master transcription factor regulating osteogenesis [27,28]. In addition, the crosstalk between BMP and TGF-β signaling via negative-feedback plays an important role during osteogenesis [29]. Therefore, we investigated the interplay between TGF-β and BMP signaling in CFC-MSCs during osteogenesis. SMAD1 is phosphorylated to form p-SMAD1 by BMP receptors. In contrast to the enhanced p-SMAD2 seen previously, decreased p-SMAD1 was observed in CFC-MSCs at d7 of osteogenic differentiation (Figure 4a). To clarify the correlation between SMAD2 and SMAD1 pathways during osteogenic differentiation, MSCs were treated with chemical inhibitors and protein agonists during osteogenesis. Activation of the SMAD2 pathway by ActA treatment of course increased p-SMAD2 level, but reduced p-SMAD1 level in WT-MSCs during osteogenic differentiation (Figure 4b). Conversely, inhibition of the SMAD2 pathway by SB-431542 (SB) treatment increased p-SMAD2 level, but elevated the p-SMAD1 level in WT- and CFC-MSCs during osteogenic differentiation (Figure 4c). These results indicate that downregulation of the SMAD1 pathway accounts for the enhanced SMAD2 pathway in CFC-Obs. Moreover, treatment with SMAD1 pathway receptor inhibitor (LDN-193189; LDN) led to activation of the SMAD2 pathway in WT-MSCs during osteogenesis (Figure 4d). However, interestingly, activation of the SMAD1 pathway by B4 treatment did not result in significant changes in p-SMAD2 levels in WT- and CFC-MSCs during osteogenic differentiation (Figure 4e). Thus, activation of the SMAD1 pathway does not seem to influence the SMAD2 pathway in the osteogenic process of MSCs.

**Figure 5:**
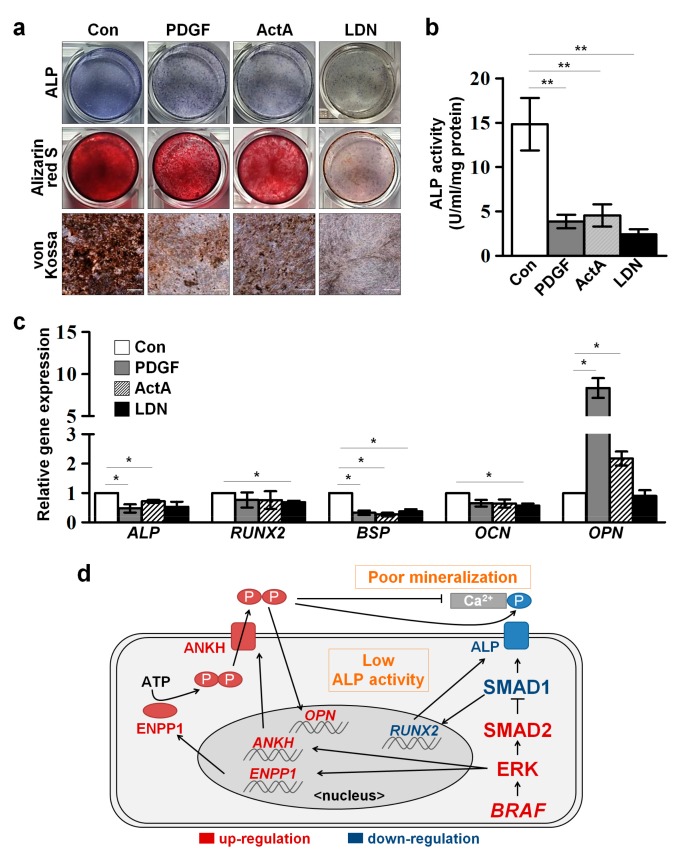
Recapitulation of osteogenic defects in WT-MSCs by regulating ERK, SMAD2, and SMAD1 signaling pathways. (**a**) Impaired osteogenesis of WT-MSCs by the signaling modulation. Activation of ERK signaling (PDGF treatment) and the SMAD2 pathway (ActA treatment), or inhibition of the SMAD1 pathway (LDN treatment) resulted in low ALP activity (upper panel) and poor mineralization as shown in Alizarin Red S staining (middle panel) and von Kossa staining (lower panel) in WT-MSCs during osteogenesis, respectively. Scale bars, 200 μm; (**b**) Reduced ALP activities in WT-MSCs by activation of ERK and the SMAD2 pathway or inhibition of the SMAD1 pathway. ALP activity was measured by using an ALP assay kit; (**c**) Transcriptional profiles of osteogenic genes in WT-MSCs after treatments with PDGF, ActA, and LDN at d7 of osteogenesis. Data were expressed as the mean ± SEM (n = 3, biological replicate). * *p* < 0.05, ** *p* < 0.01; (**d**) A schematic model for impaired osteogenesis in CFC-MSCs.

To determine whether aberrant osteogenic phenotypes can be recapitulated through signaling regulation, WT-MSCs were treated with PDGF, ActA, and LDN during osteogenic differentiation, respectively. Activation of ERK signaling (PDGF) and the SMAD2 pathway (ActA), and inhibition of the SMAD1 pathway (LDN) in WT-MSCs led to reduced ALP activity and mineral accumulation (alizarin red S and von Kossa staining) during osteogenic differentiation compared to non-treated control cells (Figure 5a,b). Transcriptional levels of osteogenic genes were changed to patterns similar to those of CFC-MSCs (Figure 5c). Collectively, our findings indicate that activated ERK signaling, an enhanced SMAD2 pathway and a decreased SMAD1 pathway, are involved in a complicated network that is associated with the impaired osteogenesis of CFC-MSCs (Figure 5d).

### 2.5. Defective Osteogenesis in CFC-MSCs Is Ameliorated by Modulation of ERK, SMAD2, and SMAD1 Signaling Pathways

**Figure 6:**
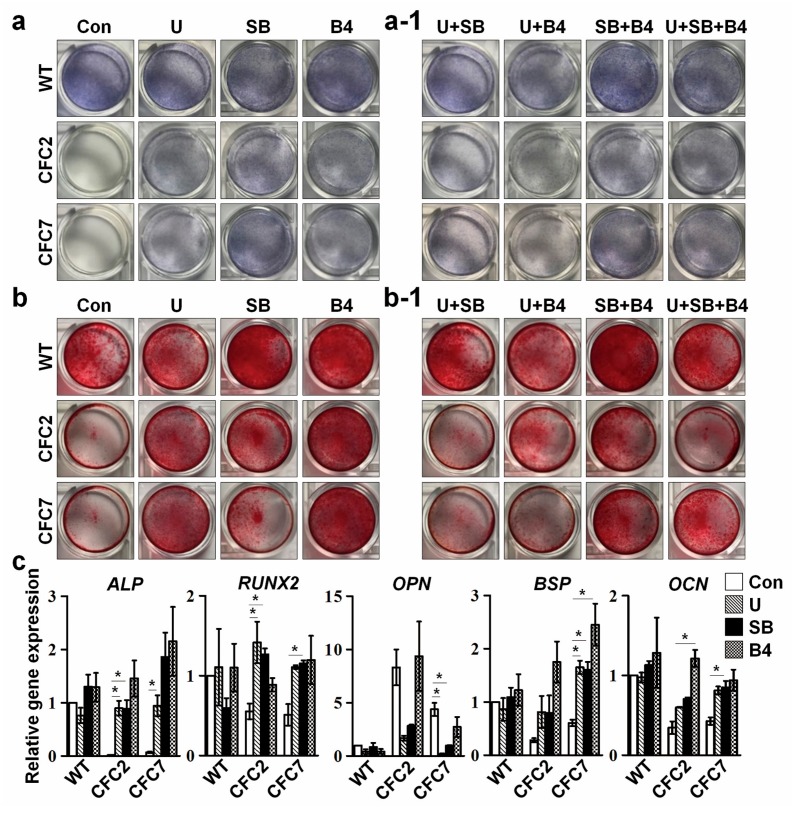
Rescue of defective osteogenesis in CFC-MSCs through regulation of ERK, SMAD2, and SMAD1 signaling pathways. ALP activity (**a**) and calcium deposition (**b**) were enhanced in CFC-MSCs during osteogenic differentiation upon treatments with an ERK signaling inhibitor (U), SMAD2 pathway inhibitor (SB), and a SMAD1 pathway agonist (B4). However, combined treatments with respective signaling regulators had no additional effects in ALP activity (**a-1**) and mineral deposition (**b-1**); (**c**) Increased expression of osteogenic genes in CFC-MSCs during osteogenic differentiation after treatments with U, SB, and B4. Data were expressed as the mean ± SEM (n = 3, biological replicate). * *p* < 0.05.

Next, experiments were performed to investigate whether controlling signaling pathways could rescue abnormal phenotypes in CFC-MSCs during the osteogenic process in vitro. Suppression of ERK signaling (U) and the SMAD2 pathway (SB) or activation of the SMAD1 pathway (B4) in CFC-MSCs recovered ALP activity (Figure 6a and Figure S3) and mineralization capacity (Figure 6b and Figure S3) to some extent in the osteogenic process. Furthermore, gene expression levels of osteogenic markers were recovered in CFC-MSCs by treatment with either chemicals to inhibit or a ligand to activate specific signaling pathways during osteogenic differentiation (Figure 6c). However, combined regulation of ERK, SMAD2, and SMAD1 signaling pathways did not exhibit synergic effects on ALP activity and mineralization in CFC-MSCs during osteogenesis (Figure 6(a-1,b-1)). Intriguingly, treatment with a RAF inhibitor (RAF-265) failed to recover the aberrant osteogenesis in CFC-MSCs (Figure S3). RAF-265 is known to be effective for cells containing the oncogenic BRAF V600E mutation, not for cells with other type of BRAF mutation [30]. A more specific approach is needed depending on the types of mutations. Next, experiments were carried out to determine whether enhanced expression of OPN in CFC-MSCs is associated with abnormal osteogenesis. It has been reported that the reduced ALP activity increases PPi level, and enhanced PPi upregulates gene expression of *OPN* [31]. Nonetheless, knockdown of *OPN* did not improve ALP activity and mineralization capacity in CFC-MSCs during osteogenesis (Figure S3). This result implies that only reducing the expression of OPN is insufficient to rescue the osteogenic defects of CFC-MSCs. Collectively, our results demonstrate that osteogenic anomalies in CFC-MSCs are ameliorated by regulating ERK, SMAD2, and SMAD1 signaling pathways (Figure 5d).

## 3. Discussion

In this study, we indicate for the first time that activation of the SMAD2 pathway as well as ERK signaling may be a critical cause of defective skeletal development in CFC syndrome patients. In CFC syndrome, ERK signaling is enhanced due to gain-of-function mutation in *BRAF*. An elevated SMAD2 pathway caused by sustained activation of ERK signaling was observed in CFC-MSCs during osteogenic differentiation in vitro. Furthermore, an enhanced SMAD2 pathway was revealed to downregulate the SMAD1 pathway in CFC-MSCs during osteogenesis. Defective ALP activity and mineralization capacity that appeared in CFC-MSCs were recapitulated in WT-MSCs during osteogenesis through activation of ERK signaling and the SMAD2 pathway or by inhibition of the SMAD1 pathway. Osteogenic abnormalities in CFC-MSCs were rescued by treatment with ERK signaling and SMAD2 pathway inhibitors and a SMAD1 pathway agonist.

The SMAD2 pathway and SMAD1 pathway play important roles in the induction of MSCs into Obs [32]. Although it is likely that activation of the SMAD2 pathway is associated with skeletal defects in various disorders, such as Marfan syndrome [33,34], osteogenesis imperfecta [35], and neurofibromatosis type-1 [36], the molecular mechanisms that explain how an enhanced SMAD2 pathway affects skeletal defects in those disorders remain unclear. Furthermore, little is known about the relevance of an enhanced SMAD2 pathway in bone formation during embryonic development in CFC syndrome. In this study, along with activated ERK signaling, CFC-MSCs showed an enhanced SMAD2 pathway and a reduced SMAD1 pathway during osteogenesis (Figure 3a and Figure 4a). The SMAD2 pathway and SMAD1 pathway are known to be regulated via negative feedback by inhibitory smads (SMAD6 and SMAD7) [37]. In CFC-MSCs, the transcript and protein levels of SMAD6 and SMAD7 were elevated at d7 of osteogenesis (Figure S4). Treatment with a SMAD2 pathway agonist (ActA) increased the level of *SMAD6* and *SMAD7* transcripts in WT-MSCs during osteogenesis (Figure S4), which is a transcriptional profile similar to that of CFC-MSCs. Furthermore, treatment with a SMAD2 pathway inhibitor (SB) reduced the level of *SMAD6* and *SMAD7* transcripts in CFC-MSCs during osteogenic differentiation (Figure S4). Enrichment of inhibitory smads has been reported to downregulate the SMAD1 pathway in osteoblastic mesenchymal cells [38]. Therefore, our results provide evidence that an enhanced SMAD2 pathway increases the expression of inhibitory smads in CFC-MSCs during osteogenic differentiation, thereby leading to downregulation of the SMAD1 pathway.

In contrast to a downregulated SMAD1 pathway during osteogenesis of CFC-MSCs (Figure 4a), increment in the SMAD1 pathway was detected in CFC-iPSCs during early neuronal development in our previous study [20]. Although ERK signaling was activated in both CFC-EBs and CFC-MSCs due to the gain-of-function mutation of *BRAF*, the activity of the SMAD1 pathway was different between CFC-EBs and CFC-MSCs. Based on these findings, it is conceivable that the SMAD1 pathway is regulated in a cell type- or tissue-specific manner in CFC syndrome during embryonic development.

The ERK signaling pathway is a critical axis in many cellular functions, including cell cycle, differentiation, and senescence. The RASopathies, a class of developmental disorders, are caused by mutations in genes that encode components of the ERK signaling pathway [39]. Regardless of the specific genetic mutations, Rasopathy patients have typical pathological features in common, such as short stature, cardiac defects, and mental retardation [40]. It has been reported that inhibition of the ERK signaling pathway is effective for alleviation of the pathophysiological phenotypes of RASopathies in animal models. For instance, treatment with ERK signaling inhibitors ameliorates cardiac and growth defects in Noonan syndrome model mice [41,42], abnormal early gastrulation in CFC syndrome model zebrafish [43], and skeletal defects and brain anomalies in neurofibromatosis type-1 model mice [44,45]. However, animal models have limitations for studying human diseases because of species specificity. In this study, we provide cellular modeling of CFC syndrome in which CFC-MSCs derived from CFC-iPSCs have a paucity of osteogenic capacity due to abnormal crosstalk between several signaling pathways, including ERK, SMAD2, and SMAD1 signaling (Figure 5d). Based on this cellular modeling, we found that aberrant osteogenesis could be recovered in CFC-MSCs by modulating the respective signaling pathways (Figure 6a–c and Figure S3). In conclusion, our findings suggest that, in addition to ERK signaling, the SMAD2 pathway and the SMAD1 pathway may be novel targets to treat CFC syndrome.

## 4. Materials and Methods

### 4.1. Ethical Statement

This study was conducted according to the principles expressed in the Declaration of Helsinki, approved by the institutional review board of Asan Medical Center (#2016-0768), and written informed consent was obtained from the parents of the patient.

All experiments using human-derived cells were conducted under the approval of the institutional review board of Korea Advanced Institute of Science and Technology (KAIST) (KH2016-52).

Animal care and experimental procedures were performed under the approval of the Animal Care Committees of KAIST.

### 4.2. Generation and Characterization of Human iPSCs

Dermal fibroblasts derived from a CFC syndrome patient were obtained from Asan Medical Center with approval by the institutional review board. Human foreskin fibroblasts (CRL-2097; ATCC, Manassas, VA, USA) were used as a WT control. CFC- and WT-iPSCs were generated from the fibroblasts, as previously described [46]. Human iPSCs were maintained in ES medium on mitomycin-C(AG Scientific Inc., San Diego, CA, USA)-treated mouse embryonic fibroblasts at 37 °C and in 5% CO_2_ in air. The ES medium consisted of DMEM/F-12 (Invitrogen, Carlsbad, CA, USA) supplemented with 20% Knockout-serum replacement (SR; Invitrogen, Carlsbad, CA, USA), 0.1 mM β-mercaptoethanol (Sigma-Aldrich, St. Louis, MO, USA), 1% penicillin-streptomycin (Invitrogen, Carlsbad, CA, USA), 1% non-essential amino acids (Invitrogen, Carlsbad, CA, USA), and 10 ng/mL fibroblast growth factor 2 (R&D Systems, Minneapolis, MN, USA). For expansion of iPSCs, respective iPSC colonies were mechanically dissected into 10–15 clumps and treated with 10 mg/mL type IV collagenase (Invitrogen, Carlsbad, CA, USA) for 4 min. Clumps were placed on fresh feeders and then cultured in ES medium for 5 d. The medium was changed daily.

One WT-iPSC line and two CFC-iPSC lines (CFC2 and CFC7) used in this study were characterized as previously reported [20,47]. For immunostaining, cells were fixed with 4% formaldehyde for 10 min and permeabilized with 0.1% Triton X-100 for 15 min. After three washes with PBST (PBS containing 0.1% Tween 20), cells were blocked with 3% bovine serum albumin (Sigma-Aldrich, St. Louis, MO, USA) at room temperature (RT) for 1 h and then incubated with primary antibodies at 4 °C overnight. The primary antibodies used in this study are listed in Table S2. After several washes with PBST, the cells were incubated with Alexa 488- or Alexa 594-conjugated secondary antibodies (Invitrogen, Carlsbad, CA, USA) at RT for 1 h. DAPI (4′,6-diamidino-2-phenylindole; Sigma-Aldrich, St. Louis, MO, USA) was used to counterstain cells for 10 min. For teratoma formation, iPSCs mixed with an equal volume of Matrigel (Corning Inc., Corning, NY, USA) were subcutaneously injected into the dorso-lateral area of CAnN.Cg-Foxn1 nu/CrljOri mice (Orient, Seongnam, Korea). Eight weeks after injection, tumor tissues were dissected and embedded into paraffin. Sectioned tissues were stained with hematoxylin and eosin and observed with a microscope (Olympus, Tokyo, Japan). The primers used for polymerase chain reaction to examine transgene expression and mutation analysis are listed in Table S3.

### 4.3. Differentiation of iPSCs into MSCs

MSC induction from iPSCs was performed as previously described [48,49,50]. Briefly, iPSC colonies were mechanically dissected into four to nine clumps (approximately 0.5 mm × 0.5 mm in size) using a tissue chopper (Mickle Laboratory Engineering Co., Surrey, UK) and detached by treatment with 10 mg/mL dispase (Invitrogen, Carlsbad, CA, USA) for 4 min. The clumps were transferred to uncoated petri dish and maintained in embryoid body (EB) medium for 1 day (d) to form EBs. The EB medium consisted of DMEM/F-12 supplemented with 10% SR, 1% penicillin-streptomycin, and 1% non-essential amino acids. EBs were cultured in suspension for 8 d in EB medium supplemented with 10 μM SB (Abcam, Cambridge, MA, USA). Then, EBs were attached on fibronectin-coated cell culture dishes and incubated in DMEM/F-12 containing 1% B27 supplement (Invitrogen, Carlsbad, CA, USA), 1% insulin-transferrin-selenium liquid media supplement (Sigma-Aldrich, St. Louis, MO, USA), 1% chemically defined lipid concentrate (Invitrogen, Carlsbad, CA, USA), and 1 μM SB. After 4 d of culture, attached cells were further incubated in α-minimum essential medium (α-MEM; Invitrogen, Carlsbad, CA, USA) supplemented with 10% fetal bovine serum (FBS) and 1% penicillin-streptomycin for 18 d. Subsequent passaging was performed with 0.25% Trypsin-EDTA (Invitrogen, Carlsbad, CA, USA) for 2 min when the cells reached 80~90% confluence.

### 4.4. FACS Analysis

MSCs were dissociated by treatment with 1 × Accutase (eBioscience, San Diego, CA, USA) for 5 min, re-suspended in fluorescence-activated cell sorting (FACS) buffer (PBS with 0.5% FBS), and then filtered using a cell strainer (SPL LifeSciences, Gyoenggi-do, Korea) with a 40-μm pore size. The antibodies used for this study are listed in Table S4. Cells were incubated with respective antibodies in dark conditions at 4 °C for 20 min and washed 3 times with FACS buffer. Finally, the cells were fixed with 10% formaldehyde solution at RT for 10 min. A minimum of 5000 cells were examined for each sample using a FACS Calibur flow cytometer (BD Biosciences, San Jose, CA, USA). Histograms for respective samples were analyzed with FlowJo software (Tree Star, Ashland, OR, USA).

### 4.5. Differentiation of MSCs into Osteoblasts, Chondroblasts, and Adipocytes

For osteogenesis, MSCs were seeded at a concentration of 2 × 10^4^ cells/cm^2^ and incubated in α-MEM supplemented with 10% FBS at 37 °C and in 5% CO_2_ in air for 3 d until confluent. Then, MSCs were cultured in STEMPRO Osteogenesis Differentiation Medium (Invitrogen, Carlsbad, CA, USA) to induce Obs. ALP activity of cells was visualized at d7 of osteogenic differentiation using a Leucocyte Alkaline Phosphatase Kit (Sigma-Aldrich, St. Louis, MO, USA) following the manufacturer’s protocol. ALP activity was quantified with a colorimetric Alkaline Phosphatase Assay Kit (Abcam, Cambridge, UK) according to the manufacturer’s protocol. For alizarin red S staining, cells at d14 of osteogenic differentiation were fixed with 10% formalin for 10 min and stained in alizarin red S solution (American Master Tech, Lodi, CA, USA) at RT for 1 h. For von Kossa staining, cells at d21 of osteogenic differentiation were fixed with 10% formalin for 10 min and incubated in 5% silver nitrate (American Master Tech, Lodi, CA, USA) at RT under ultraviolet light for 1 h. For chondrogenesis, MSCs were incubated at a high density of 1 × 10^7^ cells/mL in 10 μL of STEMPRO Chondrogenesis Differentiation Medium (Invitrogen, Carlsbad, CA, USA) on non-coated round-bottomed 96-well plates (SPL LifeSciences, Gyeonggi-do, Korea). After 1 h of incubation, 100 μL of the same medium was carefully added to each well. Spheroids aggregated from MSCs were cultured for 21 d in suspension. Chondroblasts were identified by alcian blue staining. MSC-derived spheroids were fixed with 10% formalin for 10 min and embedded in 2% agarose (LPS solution, Daejeon, Korea). Sectioned spheroids were treated with 3% acetic acid in distilled water (DW) for 3 min and incubated in alcian blue staining solution (American Master Tech, Lodi, CA, USA) at RT for 30 min. For adipogenesis, MSCs were seeded at a density of 2.5 × 10^4^ cells/cm^2^ and incubated in α-MEM supplemented with 10% FBS for 4 d until confluent. To induce adipocytes, MSCs were cultured in STEMPRO Adipogenesis Differentiation Medium (Invitrogen, Carlsbad, CA, USA) for 21 d. For oil red O staining, cells were fixed with 10% formalin for 10 min and stained in oil red O solution (American Master Tech, Lodi, CA, USA ) at 60 °C for 15 min. Images of the stained cells representing Obs, chondroblasts, and adipocytes were taken on an inverted microscope (Olympus, Tokyo, Japan).

### 4.6. RT-PCR and Quantitative RT-PCR

Total RNA was isolated from cells using easy-BLUE (iNtRON Biotechnology, Seongnam, Korea) and reverse-transcribed for cDNA synthesis using M-MLV Reverse Transcriptase (Enzynomics, Daejeon, Korea) according to the manufacturer’s protocol. RT-PCR was performed with Taq Polymerase (BioAssay, Daejeon, Korea) on a T100 Thermal Cycler (Bio-Rad, Hercules, CA, USA). Quantitative RT-PCR was carried out using premade 2 × mixture on a CFX-Connect^TM^ Real-Time detection system (Bio-Rad, Hercules, CA, USA ). The premade 2 × mixture consists of 40 mM Tris pH 8.4, 0.1 M KCl, 6 mM MgCl_2_, 2 mM dNTP, 0.2% fluorescein, 0.4% SYBR Green, and 10% DMSO. The reaction parameters for quantitative RT-PCR started with a denaturation step at 95 °C for 10 min followed by 40 cycles of 95 °C for 30 s, 60 °C for 30 s and 72 °C for 30 s, and a final elongation step at 72 °C for 5 min. The transcription level of each target gene was normalized to that of *GAPDH*. The Δ threshold cycle (Δ*C*t) value was calculated as the difference between the *GAPDH* Ct and the target Ct. Fold changes in mRNA expression between the sample and the control were determined using the formula 2^−(SΔ*C*t−CΔ*C*t)^. The primers used in this study are listed in Table S3.

### 4.7. Western Blotting

Cells were dissociated with Pro-Prep Protein Extraction Solution (iNtRON Biotechnology, Seongnam, Korea) supplemented with phosphatase inhibitor cocktail (10 mM NaF, 2 mM Na_3_VO_4_, 1 mM Na_2_P_2_O_4_) and homogenized on ice using Vibra cell VCX-750 (Sonics and Materials, Danbury, CT, USA). After centrifugation at 13,000× *g* for 30 min at 4 °C, supernatants were harvested. The protein concentration of each sample was measured with a Bradford protein assay. The samples were separated in 10% sodium dodecyl sulfate polyacrylamide gel electrophoresis (SDS-PAGE) gels, and proteins were transferred to nitrocellulose membranes (Whatman, Maidstone, UK). Then, the membranes were blocked in Tris-buffered saline with 0.05% Tween 20 supplemented with 4% skim milk or 4% BSA for 1 h at RT and incubated with each primary antibody at 4 °C overnight. Primary antibodies used in this study are listed in Table S5. After 3 washes with Tris-buffered saline with 0.05% Tween 20, the samples were incubated with a mouse or rabbit horseradish peroxidase (HRP)-conjugated secondary antibody (Santa Cruz, Dallas, TX, USA) at RT for 1 h. The membranes were developed using ECL solution (Merck Millipore, Billerica, MA, USA), and protein bands were detected using an LAS-4000 Mini biomolecular imager (Fuji Film, Tokyo, Japan). The intensity of each band was quantified with Image J software (NIMH, Bethesda, MD, USA). GAPDH-HRP (Santa Cruz, Dallas, TX, USA) was used as a control for normalization of band intensity in each experiment.

### 4.8. Transfection of OPN Small Interfering RNA

WT- and CFC-MSCs were transfected with the OPN small interfering RNA (*si-OPN*) (Bioneer, Daejeon, Korea) using Lipofectamine RNAiMAX Reagent (Invitrogen, Carlsbad, CA, USA) following the manufacturer’s protocol. Briefly, the mixture (1.5 μL of RNAiMAX Reagent and 25 μL of Opti-MEM medium) was incubated for 5 min at RT. Another mixture (0.5 μL of 10 μM siRNAs and 25 μL of Opti-MEM medium) was incubated for 5 min at RT. Then, the two mixtures were added in the same tube and incubated for 5 min at RT. The final mixture was added to each well containing 500 μL of the osteogenesis differentiation medium at a concentration of 3 pmol siRNA. After 24 h of incubation, the transfectants were cultured in fresh osteogenesis differentiation medium at 37 °C and in 5% CO_2_ in air for 7 d. The sequence of siRNAs used in this study is: si-*OPN* (forward 5′-GCAUUCCGAUGUGAUUGAUUU-3′ reverse 5′-AUCAAUCACAUCGGAAUGCUU-3′), scrambled siRNA (*si-SCR*) (forward 5′-GUUCAGCGUGUCCGGCGAUUU-3′ reverse 5′-CUGCCCGGACACGCUGAACUU-3′). The *si-SCR* was used as a control.

### 4.9. Chemicals and Growth Factors Used for Regulating Various Signaling Pathways

The concentrations of chemicals and growth factors employed to regulate signaling pathways were as follows: 5 μM U (Cell Signaling Technology, Beverly, MA, USA), 5 μM SB (Cayman Chemical, Ann Arbor, MI, USA), 100 nM LDN (Selleckchem, Houston, TX, USA), 50 nM RAF-265 (Cayman Chemical, Ann Arbor, MI, USA), 50 ng/mL recombinant human bone morphogenic protein 4 (BMP4; B4) (R&D systems, Minneapolis, MN, USA), 10 ng/mL PDGF (R&D systems, Minneapolis, MN, USA), and 50 ng/mL ActA (Peprotech, Rocky Hill, NJ, USA).

### 4.10. Statistical Analysis

Each experiment was independently repeated at least three times. All data are presented as the mean ± standard error of the mean (SEM). *p* values were evaluated by a two-tailed Student’s *t-*test using GraphPad Prism 5 (GraphPad Software, La Jolla, CA, USA). Statistical significance was denoted as follows: *, *p* ≤ 0.05; **, *p* ≤ 0.01.

## Supplementary Materials

Supplementary materials can be found at www.mdpi.com/1422-0067/18/12/2591/s1.

## Abbreviations

CFC	Cardiofaciocutaneous
ERK	Extracellular signal-regulated kinase
iPSC	Induced pluripotent stem cell
BRAF	Rapidly accelerated fibrosarcoma kinase B
qRT-PCR	Quantitative real-time polymerase chain reaction
MSC	Mesenchymal stem cell
WT	Wild-type
Ob	Osteoblast
HA	Hydroxyapatite
ALP	Alkaline phosphatase
PPi	Pyrophosphate
ENPP1	Ectonucleotide pyrophasphatase/phosphodiesterase 1
ANKH	Ankylosis human
OPN	Osteopontin
BSP	Bone sialoprotein
BMP	Bone morphogenetic protein
TGF-β	Transforming growth factor-β
RUNX2	Runt-related transcription factor 2
OCN	Osteocalcin
EB	Embryoid body
ActA	Activin A
SB	SB-431542
LDN	LDN-193189
PDGF	Platelet-derived growth factor-BB
U	U0126
B4	Bone morphogenic protein 4
si-OPN	Osteopontin small interfering RNA
si-SCR	Scrambled small interfering RNA
FACS	fluorescence-activated cell sorting
α-MEM	α-minimum essential medium
FBS	Fetal bovine serum
SEM	Standard error of the mean
