# Supplementary material for: Impaired Osteogenesis of Disease-Specific Induced Pluripotent Stem Cells Derived from a CFC Syndrome Patient
Source: Int J Mol Sci. 2017 Dec 1;18(12):2591. doi: 10.3390/ijms18122591 (PMC5751194; doi:10.3390/ijms18122591)
Supplement: Supplementary file 1 [file ijms-18-02591-s001.pdf]

Impaired osteogenesis of disease-specific induced pluripotent stem cells derived from a CFC syndrome patient

Jung-Yun Choi, Kyu-Min Han, Dongkyu Kim, Beom-Hee Lee, Han-Wook Yoo, Jin-Ho Choi and Yong-Mahn Han

Supplementary Tables

Supplementary Table 1. Clinical information of a CFC syndrome patient[1]

General information							
**Sex**	**Cardiac disorder**	**Facial malformation**	**Neck anomaly**	**Cutaneous anomaly**	**Short stature**	**Chest deformity**	**Mental retardation**
Male	Y	hypertelorism, low set ear, epicanthal folds, downslanting palpebral fissure, macrocephalic	short neck, webbed neck	sparse hair	Y	pectus excavatum	Y

Skeletal growth defects								
**Age**	**Height**		**Weight**		**IGF1[2]**		**IGFBP3[3]**	
year	cm	SD score	kg	SD score	ng/ml	SD score	ng/ml	SD score
5.5	99.7	-2.8	17	-1.3	74	-0.8	1630	-9.3
6.5	107.6	-2.2	19	-1.2	157	-0.4	1854	-2.7
7.5	113	-2.2	21	-1.2	167	-0.5	2817	-1.8
8.5	121	-1.5	23	-1.2	404	1.9	2710	-2.0
9.5	126.4	-1.4	25.6	-1.1	325	0.7	2730	-2.8

Bone densitometry[4]								
**Age**	**Spine AP (L1-L4)**		**Femur (Neck)**		**Femur (Troch)**		**Femur (Total)**	
year	g/cm^2	SD score	g/cm^2	SD score	g/cm^2	SD score	g/cm^2	SD score
10.5	0.588	-1.6	0.698	-1.4	0.540	-1.5	0.646	-1.7

[1]Diagnosed at 5.5 year

[2]IGF1: insulin-like growth factor 1

[3]IGFBP3: insulin-like growth factor-binding protein 3

[4]USA reference

Supplementary Table 2. Primary Antibodies used for immunostaining

Antibody	Species	Dilution	Source	Cat No.
OCT4	goat	1:200	Santa Cruz	sc-8628
SOX2	rabbit	1:200	Cell Signaling	#3579
NANOG	rabbit	1:200	Cell Signaling	#3580
SSEA4	mouse	1:200	Abcam	ab16287
TRA-1-60	mouse	1:200	Millipore	MAB4360
TRA-1-81	mouse	1:200	Millipore	MAB4381
NESTIN	mouse	1:200	Millipore	MAB5326
GATA4	mouse	1:200	Santa Cruz	sc-25310
Brachyury	rabbit	1:200	Abcam	ab20680

Supplementary Table 3. Primers used in this study

Gene	Forward (5′ to 3′)	Reverse (5′ to 3′)	Product Size (bp)
OCT4 Transgene	GTACTCCTCGGTCCCTTTCC	CCCTTTTTCTGGAGACTAAATAAA	unknown, 400>
SOX2 Transgene	CATGTCCCAGCACTACCAGA	CCCTTTTTCTGGAGACTAAATAAA	unknown, 400>
cMYC Transgene	AAGAGGACTTGTTGCGGAAA	CCCTTTTTCTGGAGACTAAATAAA	unknown, 400>
KLF4 Transgene	GAACTGACCAGGCACTACCG	CCCTTTTTCTGGAGACTAAATAAA	unknown, 400>
BRAF (mutation)	AAACAAGAGAGTAGATACGTCAGTTTC	TGGTAGGTAGAAAAGAGATATTTTTGG	264
GAPDH	GAAGGTGAAGGTCGGAGTC	GAAGATGGTGATGGGATTTC	226
RUNX2	TAGGCGCATTTCAGATGATG	GACTGGCGGGGTGTAAGTAA	205
OPN	ACAGCCAGGACTCCATTGAC	ACACTATCACCTCGGCCATC	196
OCN	GGCAGCGAGGTAGTGAAGAG	AGCAGAGCGACACCCTAGAC	194
ALP	GTACGAGCTGAACAGGAACA	CTTGGCTTTTCCTTCATGGT	151
BSP	CTCAGCATTTTGGGAATGGC	GTCACTACTGCCCTGAACTG	174
SMAD6	CGGTGACCTGCTGTCTCTTT	AGCGAGTACGTGACGGTTTT	169
SMAD7	GGGGGCTTTCAGATTCCCAA	ATTGAGCTGTCCGAGGCAAA	176

Supplementary Table 4. Antibodies used for FACS analysis

Antibody	Species	Fluorescence	Dilution	Source	Cat No.
CD73	mouse	PE	1:100	eBioscience	12-0739-42
CD90	mouse	APC	1:100	eBioscience	17-0909-42
CD105	mouse	APC	1:100	eBioscience	17-1057-42
CD34	mouse	APC	1:100	eBioscience	17-0349-42
CD45	mouse	FITC	1:100	eBioscience	11-9459-42
HLA-DR	mouse	APC	1:100	R&D systems	FAB4869A
PE-isotype	mouse	PE	1:100	eBioscience	12-4714-42
APC-isotype	mouse	APC	1:100	eBioscience	17-4714-42
FITC-isotype	mouse	FITC	1:100	eBioscience	11-4714-42

Supplementary Table 5. Antibodies used for western blotting

Antibody	Species	Dilution	Source	Cat No.
p-p42/44 MAPK (p-ERK1/2)	rabbit	1:2,000	Cell Signaling	#4370
P42/44 MAPK (ERK1/2)	rabbit	1:2,000	Cell Signaling	#9102
p-SMAD1/5/9	rabbit	1:500	Cell Signaling	#13820
SMAD1	rabbit	1:500	Cell Signaling	#9743
p-SMAD2	rabbit	1:500	Cell Signaling	#3108
SMAD2/3	rabbit	1:500	Cell Signaling	#3102
SMAD6	rabbit	1:1,000	Cell Signaling	#9519
SMAD7	mouse	1:1,000	Abcam	ab55493
RUNX2	rabbit	1:1,000	Cell Signaling	#12556
OPN	mouse	1:1,000	Abcam	ab69498
GAPDH-HRP	goat	1:1,000	Santa Cruz	sc-25778

Supplementary Figures

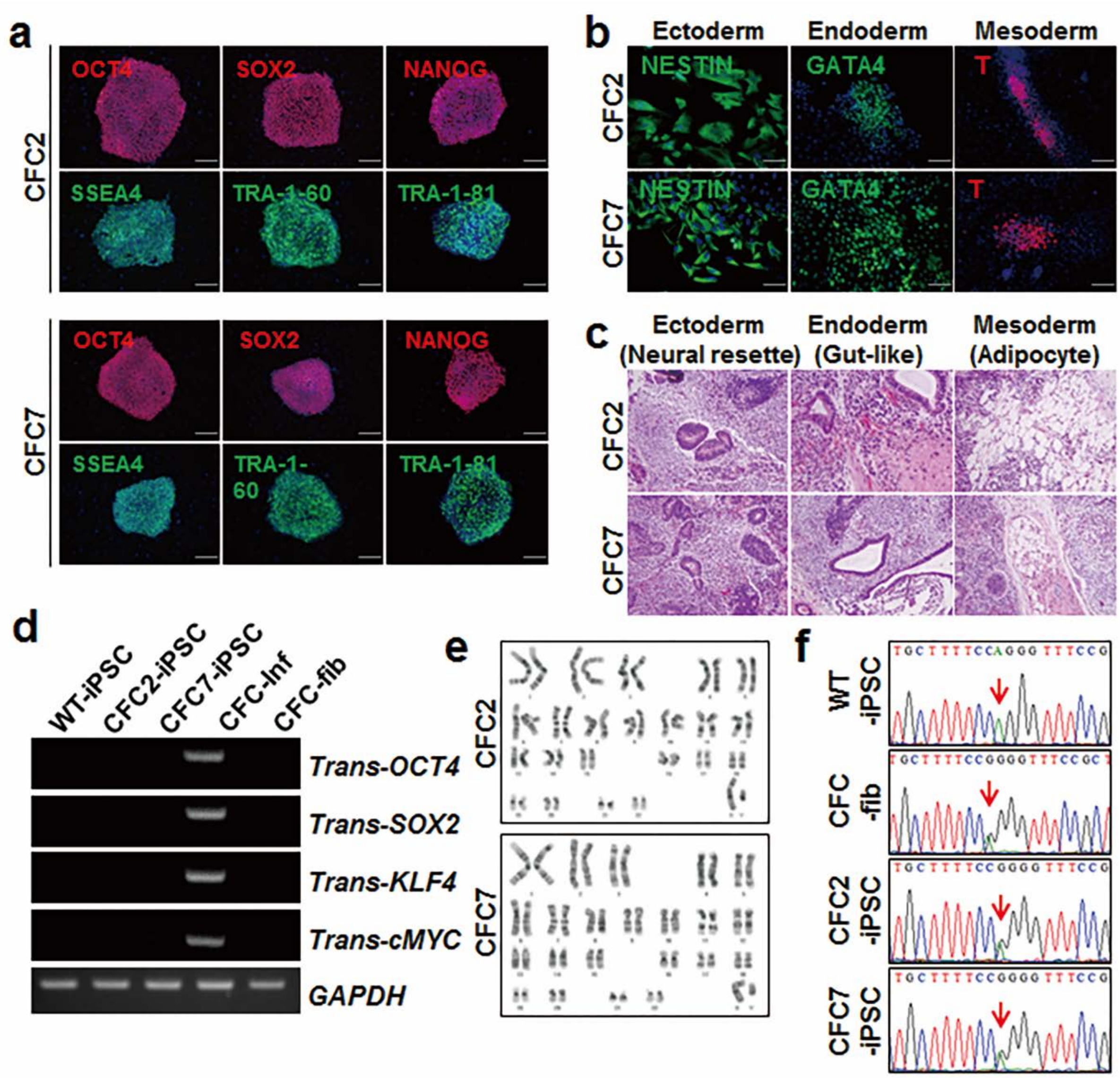

Figure S1. Generation of CFC-iPSCs. (a) Expression of pluripotent markers in CFC-iPSCs. CFC2- and CFC7-iPSCs had a normal morphology and expressed pluripotent markers. Scale bars, 200 μm. DAPI stained nuclei (blue); (b) EB formation of CFC-iPSCs. CFC-iPSCs- derived EBs were positive for representative markers of three germ layer cell types (ectoderm, NESTIN; endoderm, GATA4; mesoderm, T). Scale bars, 100 μm. DAPI stained nuclei (blue); (c) Teratoma formation of CFC-iPSCs in immunodeficient mice. Teratoma developed from CFC-iPSCs contained three germ layer cell types (ectoderm, neural rosettes; endoderm, gut-like cells; mesoderm, adipocytes). Scale bars, 100 μm; (d) No expression of transgenes (OCT4, SOX2, KLF4, cMYC) in CFC-iPSCs. Transcripts of transgenes were only detected in transgenes-infected CFC-fibroblast (CFC-inf); (e) Normal karyotype of CFC-iPSCs; (f) Genomic analysis of BRAF mutation in WT-iPSC, CFC-fib, and CFC-iPSCs. CFC-iPSCs had the same mutation with CFC-fib in BRAF gene.

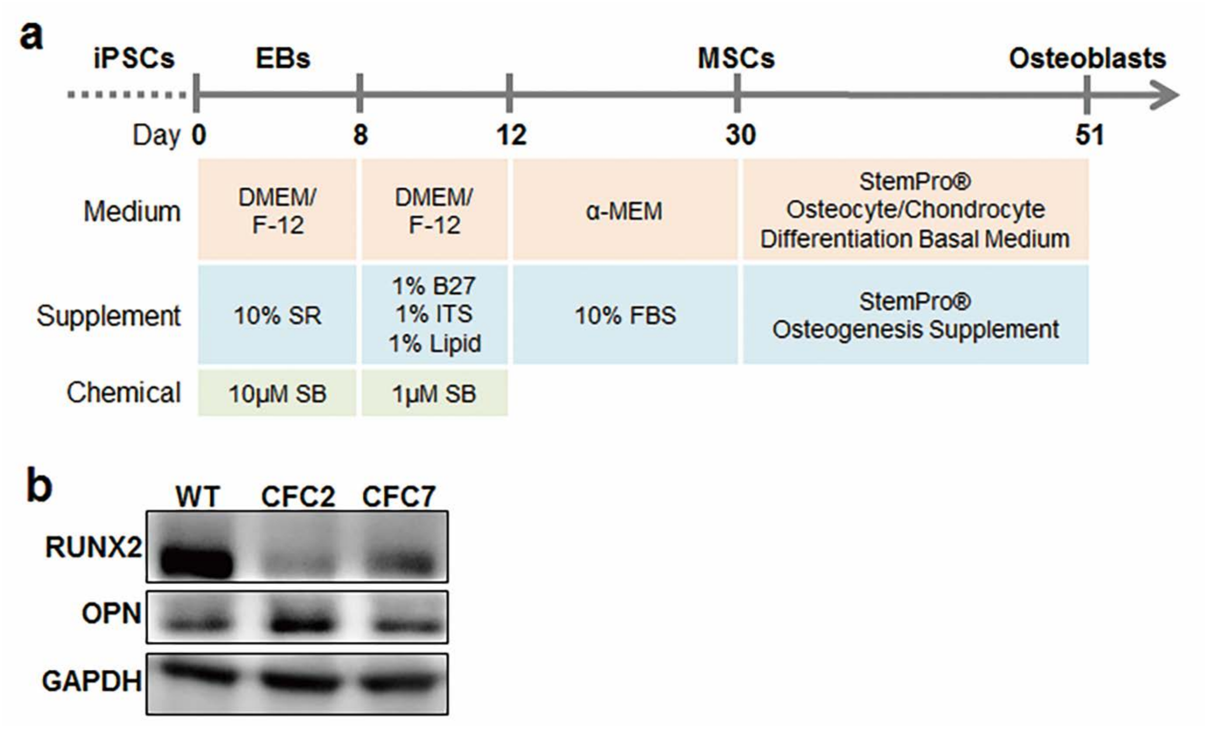

Figure S2. Differentiation scheme into osteoblasts and osteogenic protein expression in MSCs. (a) Schematic protocol for differentiation of iPSCs into Obs; (b) Downregulated RUNX2 and upregulated OPN in CFC-MSCs.

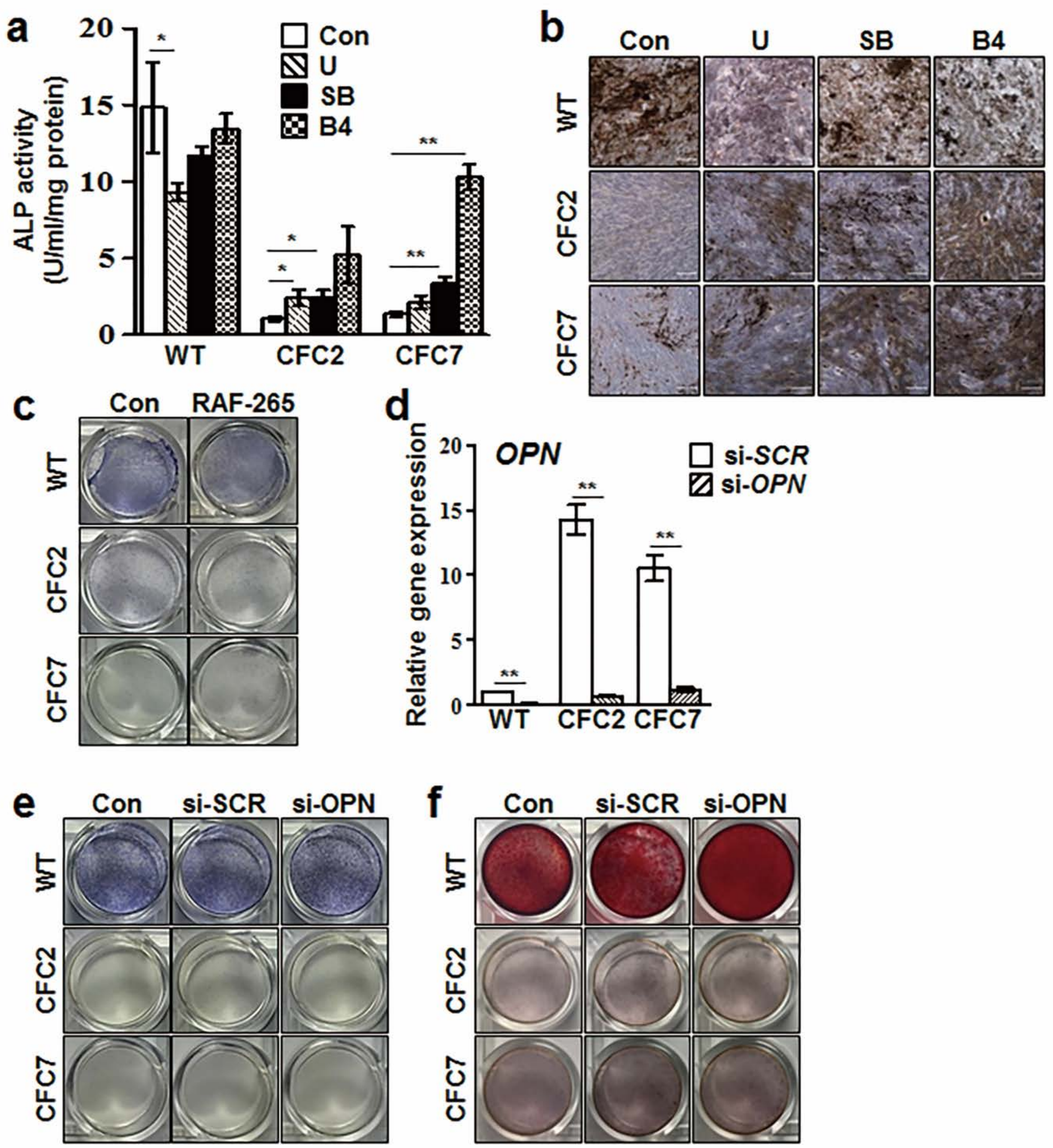

Figure S3. Effect of RAF inhibitor and OPN knockdown on the osteogenic activity in CFC-MSCs during osteogenesis. (a) Quantitative increments of ALP activity in CFC-MSCs at d 7 of osteogenesis after treatments with U, SB, and B4; (b) Improvement of calcium deposition (von Kossa staining) in CFC-MSCs during osteogenic differentiation upon treatments with U, SB, and B4. Scale bars, 200 µm; (c) ALP activity after treatment with a RAF inhibitor (RAF-265, 50 nM) in WT- and CFC-MSCs at d 7 of osteogenesis. RAF-265 did not improve ALP activity in CFC-MSCs during osteogenesis; (d) Transfection of OPN siRNA (si-OPN) in CFC-MSCs during osteogenic differentiation. Transcripts of OPN were significantly reduced in CFC-MSCs after treatment with si-OPN. Scrambled siRNA (si-SCR) was used as a control. Data were expressed as the mean ± SEM (n = 3, biological replicate). *, $p < 0.05$; **, $p < 0.01$; No effect of OPN knockdown on the osteogenic activity in CFC-MSCs during osteogenesis. Transfection with si-OPN did not improve ALP activity (e) and calcium accumulation (f) in CFC-MSCs during osteogenesis.

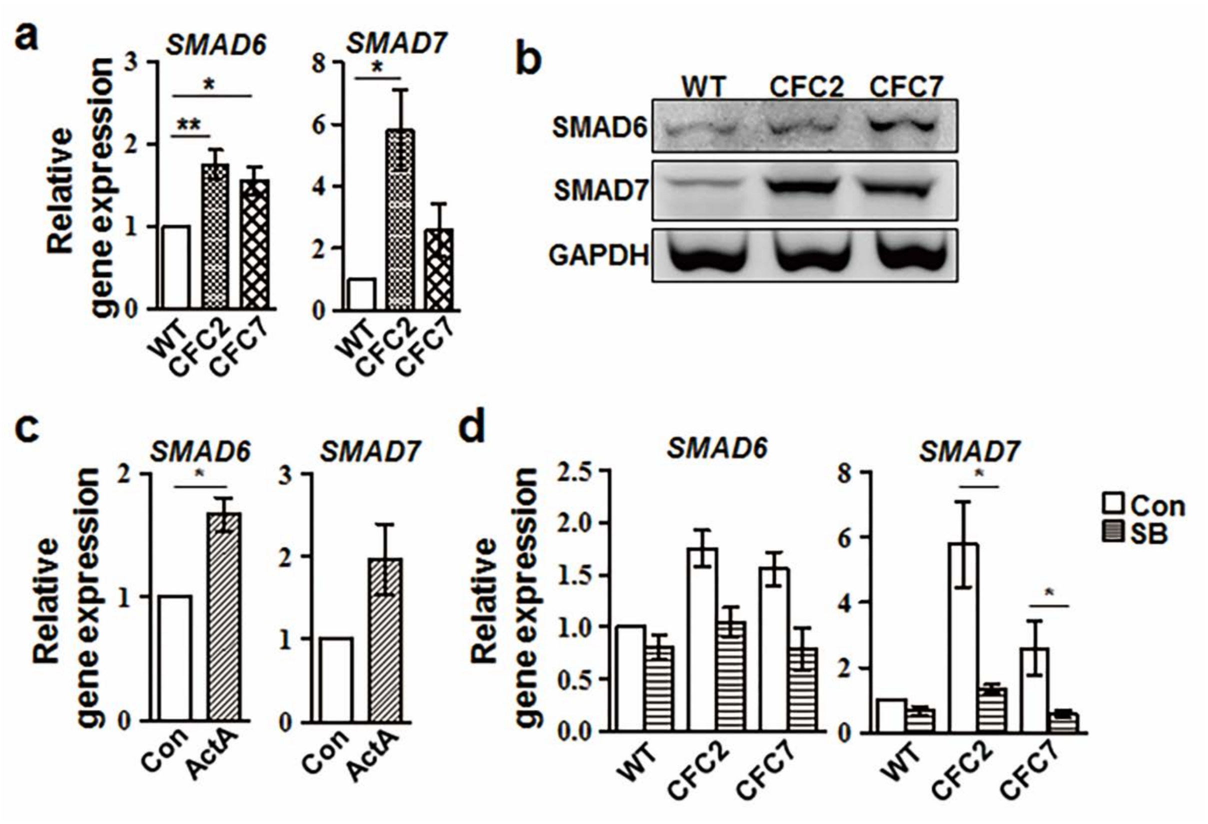

Figure S4. Correlation of inhibitory SMADs and TGF-β signaling in WT- and CFC-MSCs during osteogenesis. Enhanced expression of inhibitory SMADs (SMAD6 and SMAD7) in CFC-Obs relative to WT-Obs at the transcription (a) and protein level (b); (c) Activation of TGF-β signaling (ActA, 50 ng/ml) increased SMAD6 and SMAD7 expression in WT-MSCs at d 7 of osteogenesis; (d) Inhibition of TGF-β signaling (SB, 5 μM) decreased SMAD6 and SMAD7 expression in CFC-MSCs. Data are shown as the mean ± SEM (n = 3, biological replicate). *p < 0.05, **p < 0.01.
